# Standardized echocardiographic assessment of cardiac function in normal adult zebrafish and heart disease models

**DOI:** 10.1242/dmm.026989

**Published:** 2017-01-01

**Authors:** Louis W. Wang, Inken G. Huttner, Celine F. Santiago, Scott H. Kesteven, Ze-Yan Yu, Michael P. Feneley, Diane Fatkin

**Affiliations:** 1 Victor Chang Cardiac Research Institute, Darlinghurst, New South Wales 2010, Australia; 2 Faculty of Medicine, University of New South Wales, Kensington, New South Wales 2052, Australia; 3 Department of Cardiology, St Vincent's Hospital, Darlinghurst, New South Wales 2010, Australia

**Keywords:** Zebrafish, Echocardiography, Sex differences, Cardiac physiology, Heart failure, Regeneration

## Abstract

The zebrafish (*Danio rerio*) is an increasingly popular model organism in cardiovascular research. Major insights into cardiac developmental processes have been gained by studies of embryonic zebrafish. However, the utility of zebrafish for modeling adult-onset heart disease has been limited by a lack of robust methods for *in vivo* evaluation of cardiac function. We established a physiological protocol for underwater zebrafish echocardiography using high frequency ultrasound, and evaluated its reliability in detecting altered cardiac function in two disease models. Serial assessment of cardiac function was performed in wild-type zebrafish aged 3 to 12 months and the effects of anesthetic agents, age, sex and background strain were evaluated. There was a varying extent of bradycardia and ventricular contractile impairment with different anesthetic drugs and doses, with tricaine 0.75 mmol l^−1^ having a relatively more favorable profile. When compared with males, female fish were larger and had more measurement variability. Although age-related increments in ventricular chamber size were greater in females than males, there were no sex differences when data were normalized to body size. Systolic ventricular function was similar in both sexes at all time points, but differences in diastolic function were evident from 6 months onwards. Wild-type fish of both sexes showed a reliance on atrial contraction for ventricular diastolic filling. Echocardiographic evaluation of adult zebrafish with diphtheria toxin-induced myocarditis or anemia-induced volume overload accurately identified ventricular dilation and altered contraction, with suites of B-mode, ventricular strain, pulsed-wave Doppler and tissue Doppler indices showing concordant changes indicative of myocardial hypocontractility or hypercontractility, respectively. Repeatability, intra-observer and inter-observer correlations for echocardiographic measurements were high. We demonstrate that high frequency echocardiography allows reliable *in vivo* cardiac assessment in adult zebrafish and make recommendations for optimizing data acquisition and analysis. This enabling technology reveals new insights into zebrafish cardiac physiology and provides an imaging platform for zebrafish-based translational research.

## Abstract (precis)

**Summary:** Standardization of zebrafish echocardiography provides insights into cardiac physiology in normal and diseased states, with application for functional studies in zebrafish models of heart disease.

## INTRODUCTION

The zebrafish (*Danio rerio*) is an increasingly popular vertebrate model organism for research studies into human diseases because of their low maintenance costs, high fecundity, fast generation times and ease of genetic manipulation (Bakkers, 2011; Shih et al., 2015).

Embryonic zebrafish are highly informative for investigating cardiac developmental processes owing to the optical transparency of young fish that allows direct visualization of the heart. However, there is a progressive loss of body transparency with increasing age, and thus a rate-limiting step in using zebrafish to model adult-onset cardiovascular disorders has been a lack of tools for *in vivo* assessment of the mature heart. Recently, adult zebrafish have been shown to develop profound ventricular remodeling in response to environmental insults (Hein et al., 2015; Sun et al., 2009). These important observations point to the untapped potential of adult zebrafish for studying a broad range of human heart disorders including heritable and acquired cardiomyopathies and post-infarction myocardial regeneration.

Echocardiography is widely used in clinical practice and in mammalian animal models to assess cardiac function *in vivo* (Gueret et al., 1980; Locatelli et al., 2011; Schiller et al., 1983; Tanaka et al., 1996; Watson et al., 2004). As a non-invasive ultrasound-based imaging modality, it allows serial assessment of cardiac structure and function. Studying an aquatic organism with an adult size ranging from 20-40 mm in length is not without its challenges, but is now possible through advances in high-frequency ultrasound (up to 70 MHz, 30 µm axial resolution). Although the use of high frequency echocardiography in zebrafish has recently begun to be explored, there is a critical lack of standardized approaches for image acquisition and data analysis. Studies to date (Table S1) (Ernens et al., 2016; González-Rosa et al., 2014; Hein et al., 2015; Ho et al., 2002; Huang et al., 2015; Kang et al., 2015; Lee et al., 2014, 2016; Parente et al., 2013; Sun et al., 2008, 2015; Wilson et al., 2015) have displayed substantial differences in methodology, including scanning environment (room air versus underwater), choice and concentration of anesthetic agent, scanning views and analysis techniques, and fish age, sex and background strain, with limited data on quality control and reproducibility.

The aim of our study was to develop, optimize and validate a protocol for underwater zebrafish echocardiography under conditions as close as possible to the normal physiological state. We employed reverse translation of echocardiographic principles used in clinical practice, and have adapted these for use in a small aquatic organism. Here, we show that high-resolution imaging of adult zebrafish hearts is feasible and can provide detailed quantitative assessment of ventricular size and function. We evaluated indices of ventricular systolic and diastolic performance, and determined the effects on these parameters of anesthetic agent, age, sex, and background strain. To investigate whether echocardiography is sufficiently sensitive to detect disease-associated changes in myocardial contraction, we used two models of adult cardiac dysfunction: (1) a hypocontractile model caused by diphtheria toxin A (DTA)-induced myocarditis (Wang et al., 2011), and (2) a hypercontractile model resulting from volume overload secondary to phenylhydrazine hydrochloride (PHZ)-induced hemolytic anemia (Sun et al., 2009). Collectively, our data highlight the exciting potential of high-frequency echocardiography as a tool for comprehensive *in vivo* assessment of cardiac function in adult zebrafish.

## RESULTS

### Technical feasibility

**Fig. 1.**
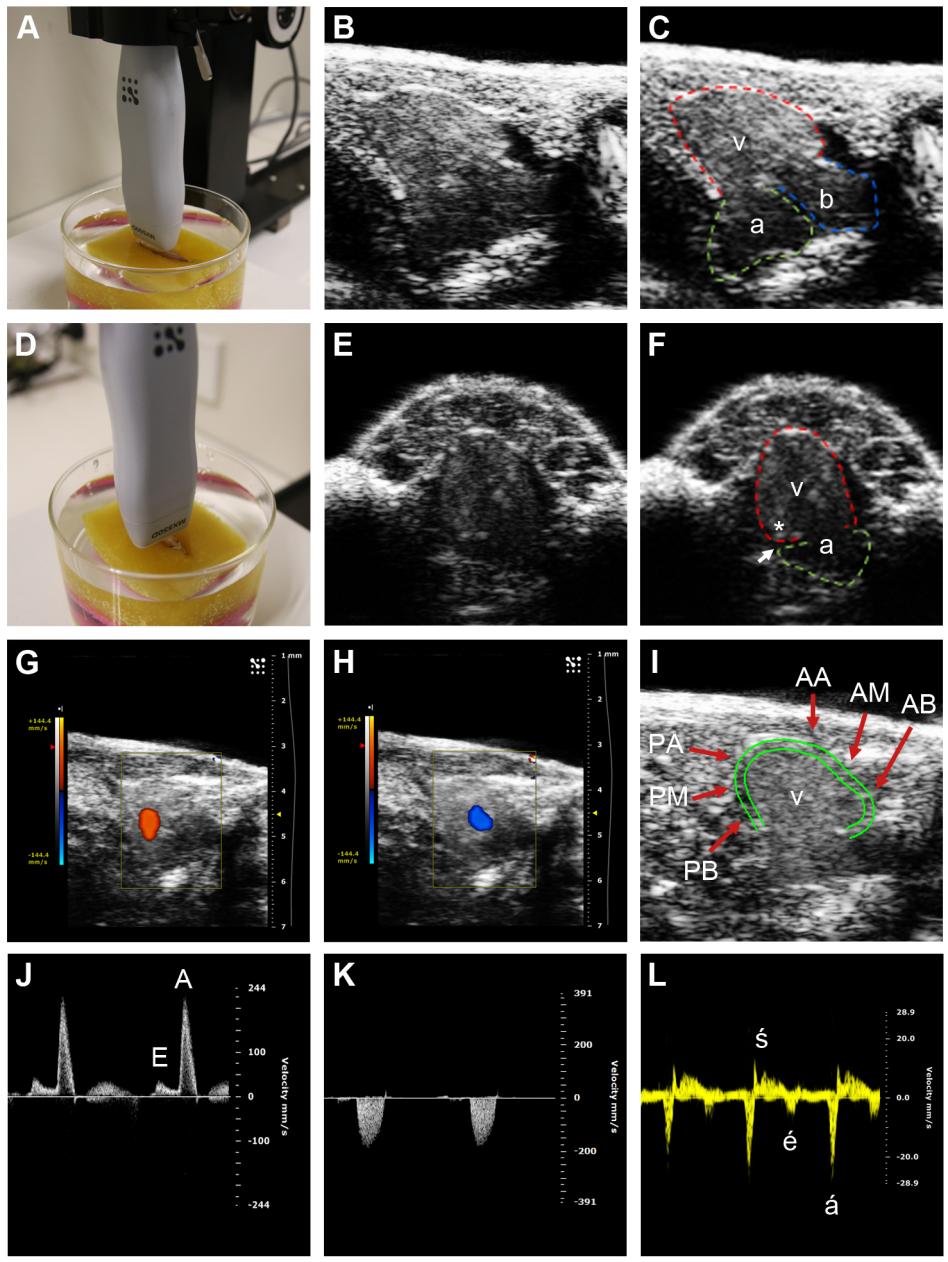
**Zebrafish echocardiographic imaging.** (A) Transducer position for the longitudinal axis (LAX) view. (B,C) LAX images, with ventricle (v, red tracing denotes epicardium), atrium (a, green tracing) and bulbus arteriosus (b, blue tracing) indicated in C. (D) Transducer position for the short axis (SAX) view. (E,F) SAX images, with ventricle and atrium indicated in F. The ventricle joins the atrium at the atrioventricular annulus, with the atrioventricular groove (white arrow) overlying this area. An asterisk labels the area of myocardium immediately adjacent to atrioventricular annulus which is the site of tissue Doppler interrogation. (G,H) Color Doppler in the LAX view showing (G) ventricular inflow (orange) from the atrium during ventricular diastole, and (H) ventricular outflow (blue) to the bulbus arteriosus during ventricular systole. (I) Ventricular wall motion was analyzed in six segments: AA, anterior (ventral) apex; AM, anterior mid; AB, anterior base; PA, posterior (dorsal) apex; PM, posterior mid; PB, posterior base. The epicardium (outer green border) and the inner border of the compact myocardium (inner green border) of the ventricle are shown. (J) Pulsed-wave Doppler interrogation of atrioventricular inflow at the level of the atrioventricular valve, showing the E (early diastolic) and A (atrial systolic) waves. (K) Pulsed-wave Doppler interrogation of ventricular outflow. (L) Tissue Doppler interrogation of the atrioventricular annulus demonstrating the é (early diastolic), á (atrial systolic) and ś (ventricular systolic) waves.

The heart was adequately visualized in all fish with high image quality (see Fig. 1 for representative images). Following a learning curve of up to 100 studies, image acquisition was typically completed within 3 min after induction of anesthesia. The procedure was well tolerated and there were no procedure-related deaths. Although imaging was technically feasible in young fish (3 months; ≥20 mm length, ≥350 mg weight), we found that superior image quality was obtained in older, larger fish (6-9 months). Image quality in female fish, especially those heavily gravid with eggs, was often lower than in males, which affected the accuracy of ventricular measurements, particularly for automated speckle tracking and strain analysis.

### Biological considerations in developing an echocardiography protocol

The zebrafish heart comprises a single atrium and ventricle, in contrast with the four-chambered hearts of reptiles, birds and mammals. The two scanning positions [longitudinal axis (LAX) (Fig. 1A-C) and short axis (SAX) (Fig. 1D-F)] were selected because they provided clear delineation of the cardiac chambers and anatomical landmarks, thus facilitating rapid and reproducible measurement. During systole, the ventricle pumps blood into the bulbus arteriosus, a reservoir from which blood empties into the ventral aorta (Fig. 1C; Movie 1). In zebrafish, the heart is located in the ventral midline, immediately caudal to the level of the gills. The apex of the heart is directed ventrocaudally. The atrium is located dorsal to the ventricle in the LAX view, whereas the bulbus arteriosus is situated dorsocranial to the ventricle (Fig. 1C).

**Fig. 2.**
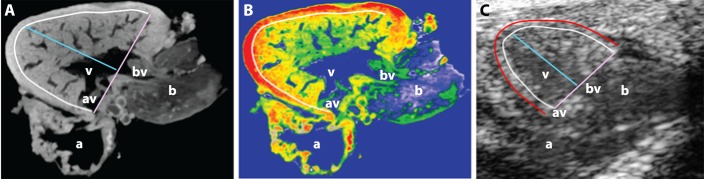
**Localization of anatomical landmarks in the zebrafish heart.** (A) Representative optical section from a three-dimensional micro-computed tomography reconstruction of a wild-type, adult zebrafish heart (Skyscan 1072, ZEISS XRadia, Belgium). (B) False-colored heat map of the section shown in A demonstrating differing densities within the ventricular myocardium. The outer compact layer possesses greater density (red) in comparison with the highly trabeculated, non-compact spongy myocardium (yellow/green). (C) Representative high-frequency echocardiography B-mode image. In all three panels, the white border demarcates the interface between the compact and non-compact layers of the myocardium. The red border in C demarcates the outer layer of the epicardium. Pink line, basal diameter (D_BASE_), the distance between anterior and posterior borders of compact myocardium; blue line, perpendicular longitudinal diameter (D_LONG_) between ventricular base to apical compact myocardial border. a, atrium; av, atrioventricular valve; b, bulbus arteriosus; bv, bulboventricular valve; v, ventricle.

Reproducible sections of the ventricle were more reliably obtained in the LAX view than in the SAX view. Consequently, B-mode images and speckle tracking in the LAX view were used to derive measurements of ventricular chamber size and function. In human heart development, there is progressive compaction of the trabeculated ventricular myocardium. Consequently, the blood–endocardial border is clearly seen and is used in clinical echocardiography to demarcate the ventricular chamber cavity. Residual trabeculation, valve leaflets and chords are included as part of the chamber cavity and not the ventricular wall for echocardiographic measurements (Rudski et al., 2010). In contrast, the mature zebrafish myocardium remains highly trabeculated (Hu et al., 2001), which complicates reproducible delineation of an endocardial border. We used the inner border of the compact myocardium, immediately adjacent to the trabeculated non-compact (‘spongy’) myocardium (inner green border, Fig. 1I) and excluded trabeculation for measurements of ventricular chamber size and contractility, analogous to standard clinical practice for measuring the relatively trabeculated human right ventricle (Rudski et al*.*, 2010). This border is clearly demonstrated by three-dimensional micro-computed tomography imaging (Fig. 2A,B) and can be discerned on high frequency B-mode echocardiography owing to changes in echogenicity arising from different tissue densities of the compacted and non-compacted myocardium (Fig. 2C). Although the epicardium (outer green border, Fig. 1I) has been used in some studies to approximate ventricular size, our B-mode and speckle-based deformation imaging showed that it undergoes relatively less displacement than the compact myocardial layer during the cardiac cycle. Consequently, epicardial-based measurements can result in underestimation of myocardial contraction (Movie 2).

Color Doppler and pulsed-wave Doppler were used for the hemodynamic assessment of ventricular inflow from the atrium during diastole (Fig. 1G,J), as well as ventricular outflow to the bulbus arteriosus during ventricular systole (Fig. 1H,K). Care was taken to ensure that the direction of the ultrasound beam was parallel to the direction of flow. Tissue Doppler assessment was performed by examining the movement of the myocardium adjacent to the atrioventricular groove during the cardiac cycle, similar to the practice of myocardial tissue Doppler assessment in humans (Fig. 1L). Doppler shift depends not only on velocity but also the angle of incidence *θ* (*Δf* is proportional to *v×cosθ*, and the maximum *cosθ*=1 when *θ=*0) and the highest measured velocity best approximates the true velocity. In the SAX view, the atrioventricular annulus was clearly discerned (Fig. 1E), and movement of the atrioventricular annulus during the cardiac cycle (ventrally during systole, dorsally during diastole) was parallel to the direction of the ultrasound beam (Movie 3), allowing more accurate assessment of tissue Doppler indices than in the LAX view.

### Comparison of different anesthetic regimens

**Fig. 3.**
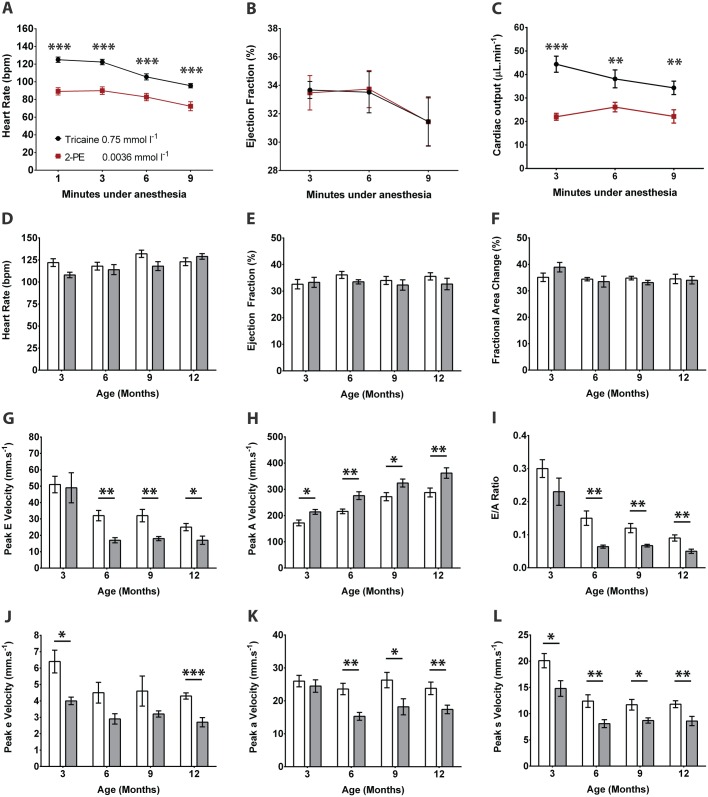
**Effects of anesthetic agents, age and sex on cardiac function.** (A-C) Male wild-type fish were anesthetized with tricaine 0.75 mmol l^−1^ (black line) or 2-phenoxyethanol 0.0036 mmol l^−1^ (2-PE; red line) (*n*=10 each group) followed by serial echocardiography over a 9-minute period. Differences in (A) heart rate, (B) ejection fraction, and (C) cardiac output were determined. (D-L) Serial echocardiography was performed at 3, 6, 9 and 12 months in male (white bars) and female (gray bars) wild-type fish (*n*=10 each group). Graphs depict (D) heart rate, (E) ejection fraction, (F) fractional area change, (G) peak E wave velocity, (H) peak A wave velocity, (I) E/A ratio, (J) peak é wave velocity, (K) peak á wave velocity, (L) peak ś wave velocity. Data shown are mean±s.e.m. *P≤0.05, **P≤0.01, ***P≤0.001, unpaired *t*-test.

Resting heart rates in adult zebrafish have been estimated to be 120-130 beats per min at 28°C (Barrionuevo and Burggren, 1999; Nemtsas et al., 2010; Verkerk and Remme, 2012). Since most anesthetic agents have negative chronotropic effects, optimizing drug selection and dose is essential in order to avoid the confounding effects of drug-induced bradycardia on measurements of cardiac chamber size and function. Two of the most commonly used agents in zebrafish studies are tricaine and 2-phenoxyethanol (2-PE). We compared these agents in male wild-type zebrafish (*n*=10 each group) and observed heart rate responses prior to echocardiographic imaging (Table S2). Using tricaine 1.5 mmol l^−1^, heart rates were (mean±s.d.) 72±15 bpm, 72±12 bpm, 71±10 bpm, and 63±10 bpm at 1, 3, 6 and 9 min after induction of anesthesia. Reducing the dose of tricaine to 0.75 mmol l^−1^ resulted in higher heart rates, particularly during the first 6 min: 125±10 bpm, 122±10 bpm, 106±11 bpm, 96±8 bpm, respectively; *P*<0.001 for all time-points versus tricaine 1.5 mmol l^−1^. With 2-PE 0.0036 mmol l^−1^, heart rates were higher than with tricaine 1.5 mmol l^−1^ (*P*<0.001) but lower than tricaine 0.75 mmol l^−1^ (*P*<0.001): 89±13 bpm, 90±14 bpm, 83±13 bpm, and 72±16 bpm at 1, 3, 6 and 9 min after anesthesia induction (Fig. 3A; Table S2). Adequate sedation for echocardiographic imaging was achieved with tricaine 0.75 mmol l^−1^ and with 2-PE 0.0036 mmol l^−1^. Lower doses of these agents resulted in inadequate or inconsistent levels of sedation and were not evaluated further.

Anesthetic agents can also have negative inotropic effects. There were no differences in ejection fraction (EF) between tricaine 0.75 mmol l^−1^ and 2-PE 0.0036 mmol l^−1^ at any time point (Fig. 3B), however 2-PE 0.0036 mmol l^−1^ was associated with a lower stroke volume at 3 min, and greater depression of cardiac output at 3, 6, and 9 min (Fig. 3C). Beyond 9 min, there were progressive reductions in heart rate and in ventricular contraction for all doses of anesthetic tested (data not shown).

In summary, tricaine 0.75 mmol l^−1^ showed the most favorable anesthetic profile, with higher heart rate and fewer cardio-depressant effects than the other drug and dose combinations tested. Given these findings, and the fact that the cardiac sonographer was able to complete all measurements within 3 min of anesthesia induction, we selected tricaine 0.75 mmol l^−1^ for all subsequent studies.

### Effects of age and sex on ventricular function

Male and female wild-type fish (*n*=10 each group) underwent serial echocardiography at 3, 6, 9, and 12 months (Fig. 3D-L; Table S3). There were no significant differences in heart rate between anesthetized males and females (Fig. 3D). Systolic function, indicated by EF (Fig. 3E) and fractional area change (FAC; Fig. 3F), did not change over time (*P*>0.05, repeated measures ANOVA) and there were no significant differences between males and females. Temporal and sex differences were found, however, in diastolic function. The peak early diastolic (E) wave velocity changed with age in in males (*P*<0.001) and females (*P*=0.001). E wave velocity was similar in both sexes at 3 months, but declined from 6 months onwards, with greater reductions seen in females (Fig. 3G). Reciprocal increases in peak atrial systolic (A) wave velocities occurred over time (*P*<0.001 for both sexes), which were more pronounced in females (Fig. 3H). These combined changes in peak E wave and A wave velocities resulted in temporal reductions in the E/A ratio in males and females (*P*<0.001), with relatively lower ratios in females at 6, 9 and 12 months (Fig. 3I). These findings suggest that compared with males, ventricular filling in female fish relies less on passive early diastolic ventricular filling and more on active atrial contraction. Tissue Doppler indices also showed significant sex differences in tissue early diastolic (é) wave (Fig. 3J), tissue atrial systolic (á) wave (Fig. 3K) and tissue ventricular systolic (ś) wave (Fig. 3L) velocities, with males having significantly higher peak values than females. In females, there was a decrease in é (*P*=0.03), á (*P*=0.01), and ś (*P*<0.001) wave velocities with increasing age. There were no statistically significant changes in é wave and á wave velocity over time in males, but the ś wave velocity decreased with age (*P*<0.001).

### Assessment of ventricular size

In clinical cardiology, ventricular chamber size is typically estimated using orthogonal diameters or derived volumes, measured at both end-diastole and end-systole. In zebrafish echocardiographic studies reported to date, ventricular size has mainly been assessed using volume, and to a lesser extent, diameter and area (Ernens et al., 2016; González-Rosa et al., 2014; Hein et al., 2015; Lee et al., 2016; Parente et al., 2013; Sun et al., 2015; Wilson et al., 2015). To evaluate methods for ventricular size estimation, we compared one-dimensional [end-diastolic longitudinal diameter (EDD_LONG_), end-systolic longitudinal diameter (ESD_LONG_), end-diastolic basal diameter (EDD_BASE_), end-systolic basal diameter (ESD_BASE_)], two-dimensional [end-diastolic area (VAd), end-systolic area (VAs)], and three-dimensional [end-diastolic volume (EDV), end-systolic volume (ESV)] parameters in wild-type zebrafish. The one-dimensional parameters, particularly EDD_LONG_ and ESD_LONG_, consistently had the smallest coefficients of variation, while EDV and ESV had the greatest variability (Table S4).

### Effects of age, sex, and body size on ventricular size

**Fig. 4.**
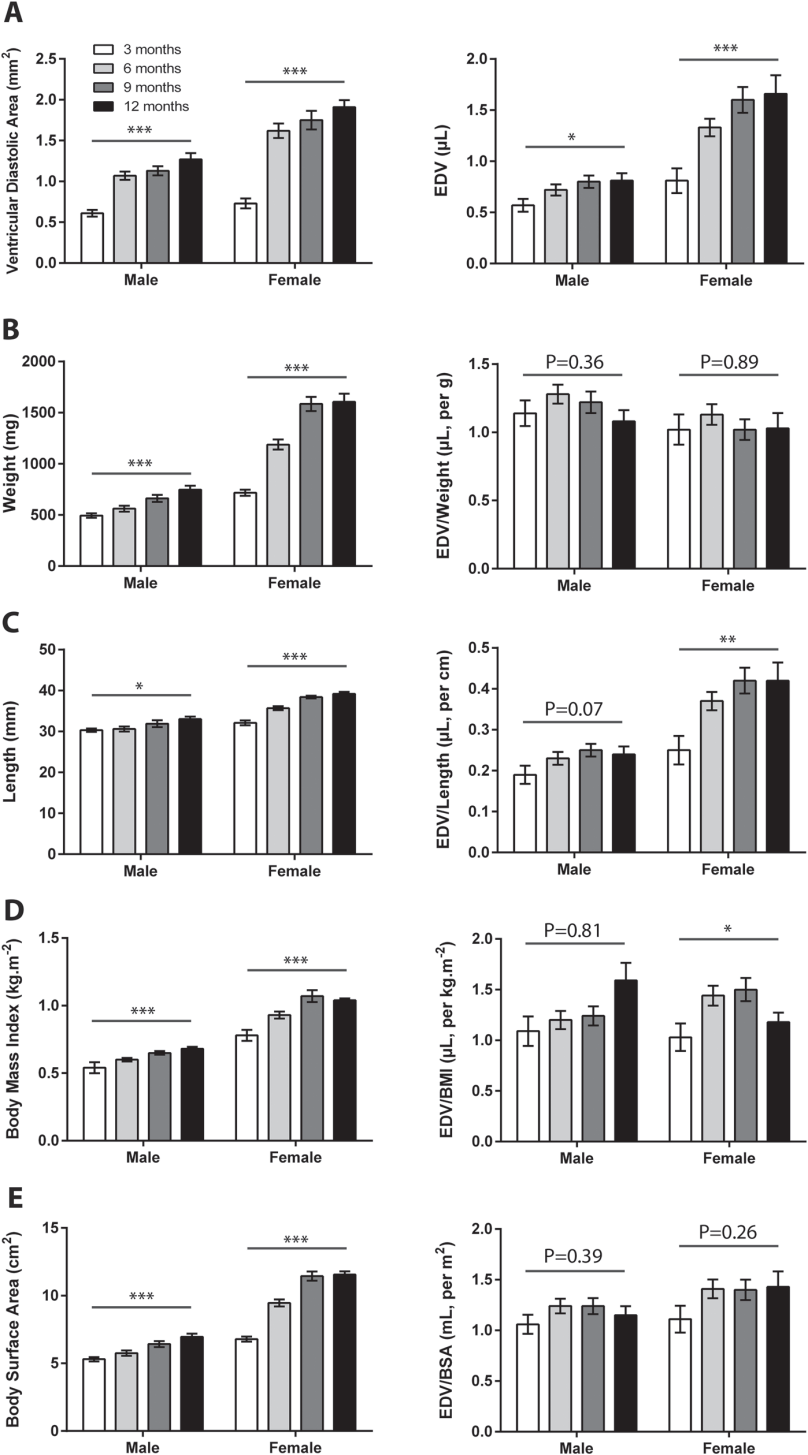
**Standardization of ventricular dimensions.** (A) Serial analysis of male and female wild-type fish (*n*=10 each group) at 3, 6, 9 and 12 months showed temporal increases in ventricular end-diastolic area (left graph) and end-diastolic volume (EDV, right graph). (B-E) Left-hand bar graphs show effects of age and sex (left columns) on (B) weight, (C) length, (D) body mass index, and (E) body surface area. Right-hand bar graphs columns show EDV normalized to each of these parameters. Data shown are mean±s.e.m. *P≤0.05; **P≤0.01; *** *P*≤0.001, (repeated measures ANOVA across four time points for each group).

The substantial variability in EDV and ESV could result from the fact that ventricular volumes are not directly measured, but derived from one-dimensional and two-dimensional data. We also considered the possibility that body size might be a confounding factor and that normalization of volumes might be required. Corresponding with normal growth, weight and body length (snout to base of tail fin) increased in all fish from 3 to 12 months of age (*P*<0.001). When compared with males, female fish were significantly heavier (Fig. 4B) and longer (Fig. 4C) at all time points, with higher body mass index (BMI; Fig. 4D) and body surface area (BSA; Fig. 4E). This was especially notable after 3 months, corresponding with the onset of gravidity in females (Table S4).

For males and females, ventricular chamber diameters significantly increased with increasing age (*P*<0.001). Ventricular areas and volumes were similar in male and female fish at 3 months, but were higher in females at 6, 9 and 12 months (Fig. 4A; Table S4).

We indexed EDV to weight (Fig. 4B), length (Fig. 4C), BMI (Fig. 4D) and BSA (Fig. 4E) in the same male and female fish at different age time-points (Table S5). With regard to sex differences, we found that indexing to weight, BMI and BSA abolished differences in EDV between male and female fish of the same age, whereas indexing to length did not. With regard to fish of different ages, EDV differences were no longer significant after indexing for BSA and weight, but remained significant for BMI and length. For EDV (and VAd), normalization to BSA and weight consistently reduced the coefficient of variation compared with that for non-standardized values, especially in male animals. Normalization did not have any noticeable effect on the coefficient of variation for the one-dimensional parameters (e.g. EDD_LONG_) but these still had the smallest coefficients of variation irrespective of normalization.

### Effects of background strain on cardiac function parameters in wild-type zebrafish

Characteristics of 9-month-old male fish (*n*=10-13) from four separate wild-type strains (*TE*, *AB*, *SUN*, *WIK*) are reported in Table 1. There were significant effects of background strain with respect to length, weight and heart rate (*P*<0.001 for all comparisons). Ventricular EDV differed between wild-type strains (*P*<0.001), even after normalization to BSA (*P*=0.007). Although EF was similar (*P*=0.15), the results for global longitudinal strain (GLS) suggest that there might be subtle inter-strain differences in baseline systolic function. There were significant differences in pulsed wave Doppler and tissue Doppler indices indicating likely inter-strain effects on diastolic function.

**Table 1.**
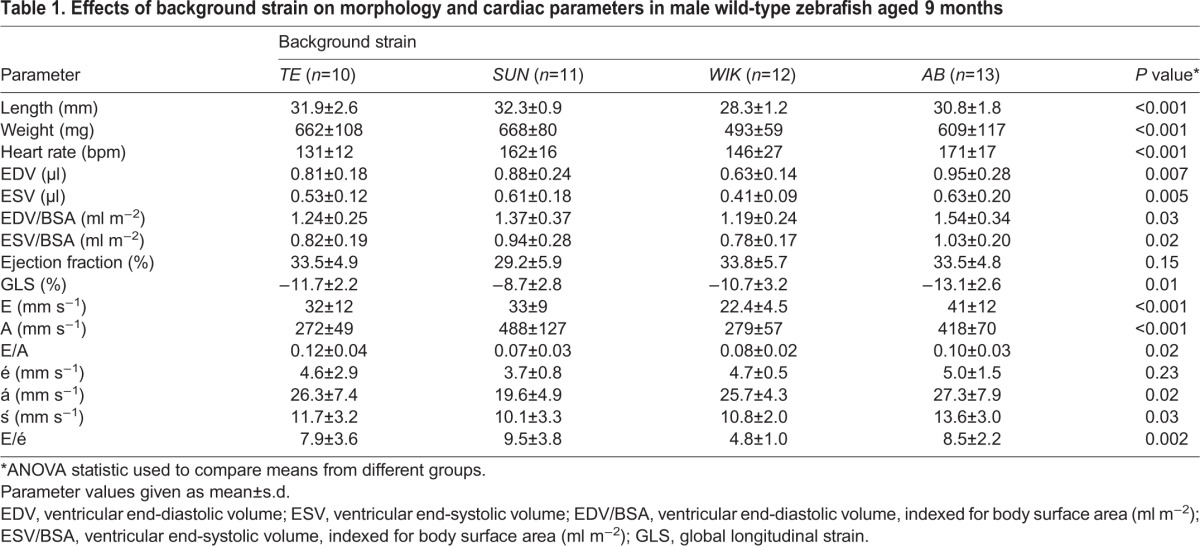
**Effects of background strain on morphology and cardiac parameters in male wild-type zebrafish aged 9 months**

### DTA-mediated cardiomyocyte loss impairs ventricular contractile function

**Fig. 5.**
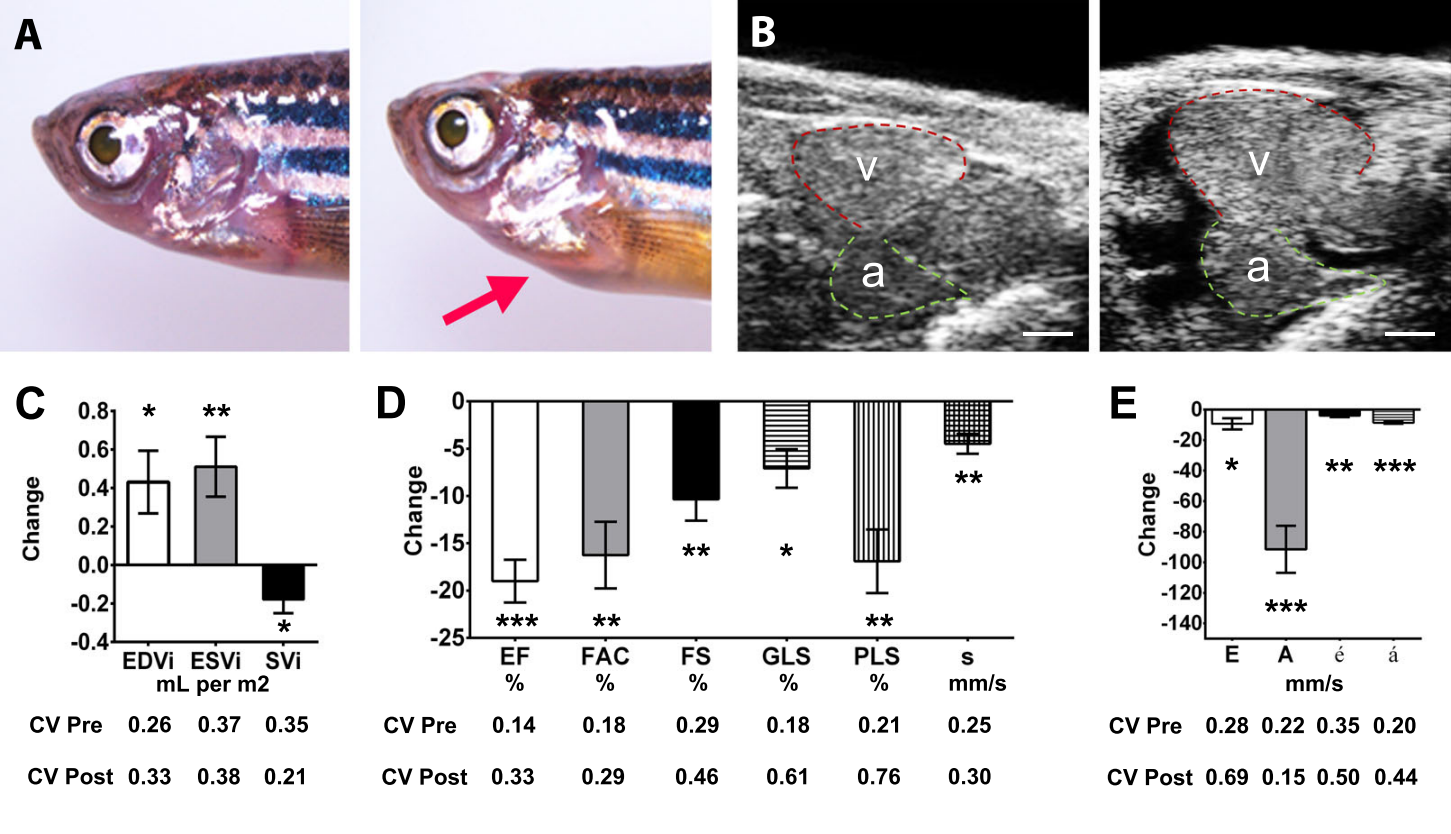
**Diphtheria toxin A (DTA) myocarditis model.** Serial echocardiography was performed in male wild-type fish (*n*=8) to assess the effects of conditional cardiomyocyte-specific expression of DTA by 4-hydroxy-tamoxifen (4-HT) exposure. (A) Macroscopic appearance before (left) and 7 days after (right) 4-HT exposure. Treated fish show ventral protrusion (arrow) associated with pericardial effusion and cardiac chamber enlargement. (B) Echocardiographic longitudinal axis images showing ventricle (v, outlined in red) and atrium (a, outlined in green) before (left) and after (right) 4-HT exposure. Scale bar: 0.2 mm. (C) Bar graph depicting mean absolute change in end-diastolic volume indexed to BSA (EDVi), end-systolic volume indexed to BSA (ESVi) and stroke volume indexed to BSA (SVi). (D) Bar graph depicting the mean absolute change from baseline for indices of systolic function: ejection fraction (EF), fractional area change (FAC), fractional shortening (FS), global longitudinal strain (GLS), peak longitudinal strain (PLS) and peak ś wave velocity on tissue Doppler. (E) Bar graph depicting mean absolute change in parameters of diastolic function: peak E wave, A wave, é wave and á wave velocities. For parameters shown in C-E, the coefficient of variation (CV, where CV=s.d./mean) was calculated for pre-treatment and post-treatment measurements. Data are shown as mean±s.e.m. *P≤0.05; **P≤0.01; ***P≤0.001, paired *t*-test.

To determine whether pathological myocardial contractile impairment could be effectively identified by echocardiography, we evaluated cardiac function in male wild-type fish (*n*=8) before and 7 days after induction of DTA by 4-hydroxy-tamoxifen (4-HT) (Fig. 5; Tables S6, S7; Movies 4, 5). The 4-HT-treated fish typically developed a ventral protrusion (Fig. 5A) associated with pericardial effusion and cardiac chamber dilatation (Fig. 5B,C). There were significant reductions in EF and in all other indices of ventricular systolic contraction (Fig. 5D). Despite a preserved E/A ratio, there was clear evidence of diastolic dysfunction, with reductions in peak E, A, é, and á wave velocities, indicative of impairment of both ventricular relaxation and atrial systolic contraction (Fig. 5E). 4-HT treatment was associated with increased myocardial wall thickness (mean increase 0.18±0.10 mm, *P*=0.001), consistent with edema resulting from cardiomyocyte cell death, which is frequently observed echocardiographically in humans affected by acute fulminant myocarditis (Felker et al., 2000). There was a high correlation between the reduction in EF (mean decrease −0.19±0.06, *P*<0.001) and the extent of cardiomyocyte loss (72±13%, r=0.93, *P*=0.003; Fig. S1).

### Anemia-induced volume overload results in ventricular hypercontractility

**Fig. 6.**
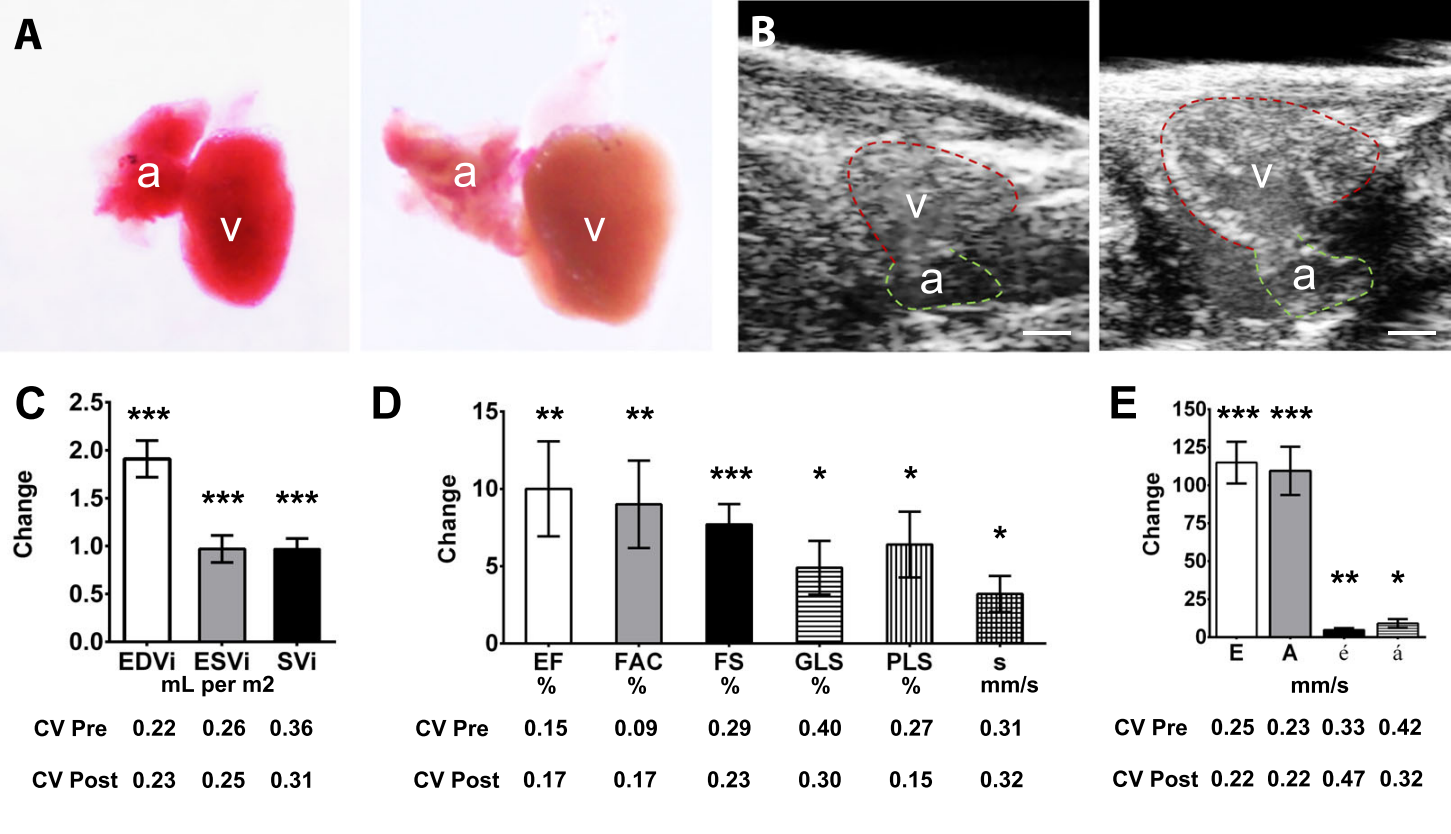
**Chronic anemia model.** Serial echocardiography was performed in male wild-type fish (*n*=8) to assess cardiac function before and after an 18-day phenylhydrazine hydrochloride (PHZ) treatment protocol to induce hemolytic anemia. (A) Representative explanted hearts from an untreated fish (left) and a fish treated with PHZ (right); treated hearts developed pallor and enlargement of the ventricle (v) and atrium (a). (B) Longitudinal axis images showing ventricular and atrial size before (left) and after (right) PHZ. Scale bar: 0.2 mm. (C-E) Description of bar graphs depicted in C-E as for Fig. 5. Data are shown as mean±s.e.m. *P≤0.05, **P≤0.01; ***P≤0.001 (paired *t*-test).

Sustained treatment of adult zebrafish with PHZ has been shown to induce chronic hemolytic anemia and cardiac remodeling associated with cardiomyocyte hypertrophy and hyperplasia (Sun et al., 2009). In human hearts, chronic anemia causes a high cardiac output state and ventricular hypercontractility, but whether these changes are recapitulated in the zebrafish heart has not been evaluated. We performed echocardiographic studies in male wild-type zebrafish (*n*=8) at baseline and after an 18-day period of PHZ administration every second day (Fig. 6; Tables S6, S8; Movies 6, 7). PHZ-treated fish showed generalized pallor, which was also observed in the explanted hearts (Fig. 6A), with ventricular dilatation (Fig. 6B,C), increases in EF and all other indices of ventricular systolic contraction (Fig. 6D), and increased cardiac output. There were increases in all diastolic inflow and tissue velocities (Fig. 6E). The net increase in the E/A ratio suggests that in volume-overloaded hearts, a greater proportion of ventricular filling results from early passive filling resulting from enhanced ventricular relaxation.

### Comparison of cardiac functional parameters

Echocardiography provides a number of different parameters of ventricular systolic and diastolic function, but optimal indices for cardiac function assessment in adult zebrafish have yet to be determined. To address this question, we evaluated the relative extent of change and variability in B-mode, pulsed-wave Doppler, and tissue Doppler parameters in our two models of acquired myocardial dysfunction. Variability was quantified using the coefficient of variation (s.d./mean). In general, there was more variability in the post-treatment measurements than in baseline data, reflecting not only intrinsic differences between animals but also differential responses to treatment.

For the derived systolic parameters, the best indices with respect to high net change and small variability were EF and FAC (Fig. 5D, Fig. 6D). In each of our two disease models, it is notable that the major indices of systolic function showed parallel directions of change, indicative of hypocontractility and hypercontractility, respectively. A comparative segmental strain analysis showed that longitudinal strain parameters were more consistently altered than radial parameters. This is in keeping with the observation that in the LAX view, contraction of the zebrafish ventricle occurs from the base to the apex predominantly in a direction parallel to the plane of the axis, rather than radial shortening (Movie 2). Global and peak values for longitudinal strain, strain rate and displacement had less variability than individual segment values (Fig. S2, Tables S7, S8). Consistent with our observation that the epicardium in zebrafish does not undergo the same degree of displacement as the endocardium during the cardiac cycle, estimation of FAC using the inner border of the compact myocardium detected larger changes in systolic function than epicardial FAC, with a greater net change and statistical significance in both disease models (Table S6).

For diastolic parameters, higher velocity signals tended to show less variability than low velocity signals, with the peak A wave velocity showing the highest net change and lowest variability across both models (Fig. 5E, Fig. 6E). As for the systolic variables, there was a concordance in the direction of change for all the diastolic variables in both the DTA and chronic anemia models.

### Reproducibility of echocardiographic data

To determine intra-observer variability and repeatability of echocardiographic measurements, 10 male wild-type fish were scanned twice, at a 5-day interval, with image acquisition and data analysis undertaken by a single observer. When the first set of images was analyzed twice by the same observer, the mean difference in EF was −0.02% (95% CI −2.4 to 2.4%) with an intra-class correlation coefficient for single measures of 0.92 (95% CI 0.82 to 0.97). The average measures intra-class correlation coefficient was 0.96 (95% CI 0.90 to 0.98), suggesting that there was high reproducibility in mean EF measured by the same operator in the same population. For two-dimensional area measurements, short axis length, pulsed-wave Doppler and tissue Doppler indices, the intra-class correlation coefficients were very high (0.98 to 1.00) (Table S9).

When the first and second set of images were both analyzed by the same observer, the mean difference in EF between two measurements was −0.02% (95% CI −3.3 to 3.3%), with an intra-class correlation coefficient for single measures of 0.90 (95% CI 0.77 to 0.96). The average measures intra-class correlation coefficient was 0.95 (95% CI 0.87 to 0.98), suggesting that there was relatively high reproducibility in mean EF measured in the same population on different days. For other parameters, the agreement between the two sets of measurements was very high with intra-class correlation coefficients ranging from 0.97 to 0.99 (Table S9).

To evaluate inter-observer variability, the first set of echocardiographic images was analyzed by two independent observers. Reproducibility for EF was moderate (single measures inter-class correlation coefficient 0.74, 95% CI 0.46 to 0.89, average measures inter-class correlation coefficient 0.85, 95% CI 0.63 to 0.94). For other parameters, the inter-class correlation coefficients remained moderately high, particularly with regards to the average measures inter-class correlation coefficient (Table S9). Thus, although inter-observer variability was greater than intra-observer variability for individual measurements, there remained moderately high reproducibility in the mean population measurements.

### Power calculations

Animal and operator variability and intervention effect size directly influence the sample size needed to sufficiently power studies to reliably detect statistically significant differences when present. To estimate the numbers of fish required for zebrafish echocardiographic studies, we undertook power calculations based on data from our group and others for standard deviations in EF in wild-type fish (Table S10). For s.d. of 5%, experimental models expecting large changes in EF require few fish to achieve sufficient power. However, study groups of ≥10 fish per investigation arm are required to detect absolute changes in EF of ∼5% in a paired study design and ≥17 fish per investigation arm in an unpaired design. Experiments expecting higher s.d, or smaller differences in mean EF would require substantially greater numbers of fish per investigation arm for adequate power.

## DISCUSSION

Here, we present a comprehensive evaluation of underwater high-frequency echocardiography for assessment of cardiac function in adult zebrafish. A number of technical and biological factors were identified that influence image quality and data analysis, and different indices of cardiac systolic and diastolic function were compared. Echocardiography provided novel insights into zebrafish cardiac physiology, and was able to successfully discriminate between normal heart function and pathological states. Our data enable us to make recommendations for undertaking echocardiographic studies in adult zebrafish (Table 2) and for study design.

**Table 2.**
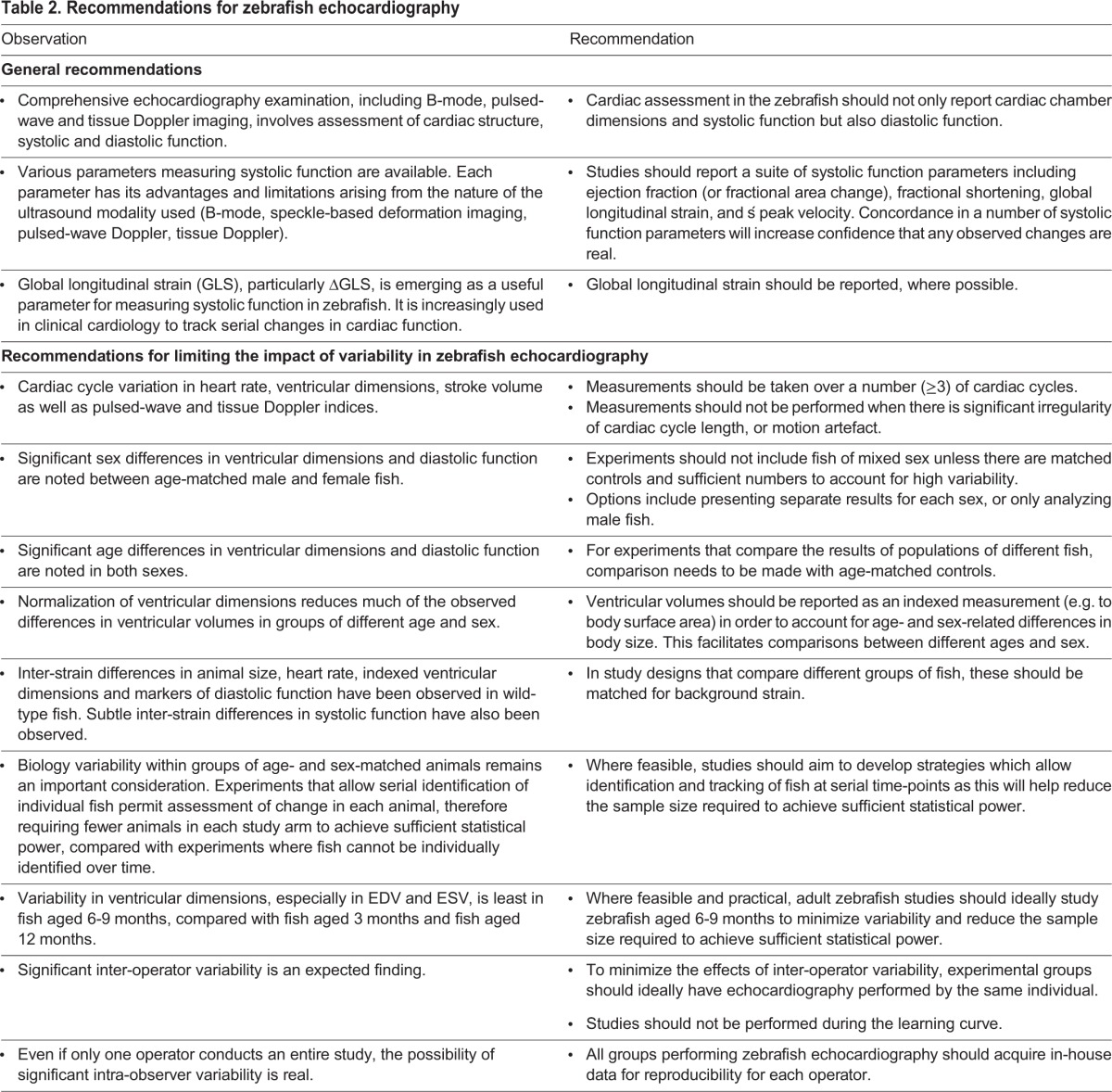
**Recommendations for zebrafish echocardiography**

In order to obtain meaningful functional information, echocardiographic imaging ideally needs to be performed under conditions that closely approximate the normal physiological state. Important factors that can profoundly influence heart rate and contractile function include the imaging milieu, anesthesia and environmental temperature (Barrionuevo and Burggren, 1999; Huang et al*.*, 2010). In previous work, both underwater and out-of-water techniques have been used (Ernens et al., 2016; González-Rosa et al., 2014; Hein et al., 2015; Ho et al., 2002; Huang et al., 2015; Kang et al., 2015; Lee et al., 2014, 2016; Parente et al., 2013; Sun et al., 2008, 2015; Wilson et al., 2015). Since zebrafish are aquatic organisms, underwater scanning has distinct advantages for *in vivo* imaging including maintenance of normal gill ventilatory activity and gas exchange. Temperature, water characteristics and hydrostatic pressure can all affect oxygen concentration and are more readily controlled in an underwater setting (Barrionuevo and Burggren, 1999). Additionally, underwater scanning obviates the need for ultrasound gel owing to the excellent ultrasound­-conducting properties of water and is overall likely to be less stressful for fish than out-of-water methods.

Although anesthesia is necessary to allow immobilization and positioning of fish, drug-related reductions in heart rate and myocardial contractility can confound the assessment of cardiac phenotypes. Tricaine and 2-PE have been previously used to induce and maintain anesthesia in zebrafish (Table S1). We observed progressive bradycardia and reduced EF with both agents over a range of doses and found that tricaine 0.75 mmol l^−1^ gave the most favorable profile while at the same time providing adequate anesthesia and respiration. Given the marked decline in functional parameters after 6 min of anesthesia, information obtained after this time is unreliable and image acquisition should ideally be completed within 3 min. Fish that are physiologically compromised as a result of disease (e.g. heart failure) display a significant time-dependent decrement in heart rate and are likely to be more susceptible to the cardio-depressant effects of anesthesia (Wang et al., 2016). This is an important consideration in zebrafish disease models evaluating heart rate and cardiac function. There are a number of other options for zebrafish anesthesia, including gradual cooling, isoflurane, metomidate hydrochloride and ketamine. These methods each have their limitations and were deemed less suitable for high-throughput studies than the agents we selected for testing. Future refinements of anesthetic protocols using different agents or combinations might help to minimize adverse effects and permit longer imaging times.

Fish age, sex and background strain were found to be significant variables in cardiac function analysis. Female fish were technically more difficult to scan underwater than male fish as their lipid-rich eggs added buoyancy, which created movement artefacts and reduced image quality. Indices of systolic function were similar over time and between sexes. However, we found a divergence between sexes in diastolic function that became apparent with increasing age. When compared with males, female fish had lower peak E wave velocity from 6 months of age, with correspondingly higher peak A wave velocities. These observations are consistent with females having lower ventricular compliance than age-matched males, a finding similarly noted in humans (Hayward et al., 2001). Our study did not investigate the molecular mechanisms underlying sex differences in diastolic function in zebrafish, but there are a number of possible factors, including age-related changes in myocardial collagen content, sarcomere protein expression levels and post-translational modifications. At any given age, female fish were much larger than male fish raised in identical conditions (feeding, tank density, temperature). Although female fish had larger ventricular chamber dimensions than males, there were no sex differences in ventricular size when these measurements were normalized to body size (see below). We also observed inter-strain differences in morphology, heart rate and cardiac function parameters in male wild-type zebrafish aged 9 months, a time-point when a majority of lifetime growth has occurred and both sexes are fertile. Taken together, these data suggest that studying a single sex might be preferable to mixed populations, depending on the research question under consideration, as this is likely to minimize both variability and the numbers of animals required to detect statistically significant differences. Standardizing background strain is an important additional consideration in experimental design.

There are a number of options for estimating ventricular size. Of the various parameters assessed, we found that variability was least with one-dimensional parameters (e.g. EDD_LONG_), and highest with three-dimensional parameters (e.g. EDV and ESV). The variability in ventricular volumes was maximal in zebrafish at the extremes of the ages we studied, and might be explained in young fish by problems of finite resolution when imaging small ventricles, and in older fish by variations in body size. The more modest variability in volumes seen at 6-9 months suggests that this might be an ideal age range for undertaking adult zebrafish studies. Given these variability considerations, ventricular size seemed to be best assessed by measurement of linear diameters, especially EDD_LONG_ and ESD_LONG_. However, EDV and ESV need to be determined as these are required for calculation of EF. It is useful to provide these complementary types of measurements, as any changes in ventricular size would be more confidently identified by concordant trends in these data. To account for individual body habitus, indexation of ventricular size is common practice in clinical echocardiography (Rudski et al., 2010). Accordingly, we found that normalizing for BSA and weight facilitated standardized comparison of ventricular volume between groups of zebrafish of different age and sex. There were negligible effects of normalization on chamber diameters, as these already had small coefficients of variation.

EF is a commonly used parameter of ventricular systolic function in humans and rodents. In wild-type zebrafish, reported values for EF have ranged from <30% to >50% (Ernens et al., 2016; González-Rosa et al., 2014; Hein et al., 2015; Ho et al., 2002; Huang et al., 2015; Kang et al., 2015; Lee et al., 2014, 2016; Parente et al., 2013; Sun et al., 2008, 2015; Wilson et al., 2015). Several factors might contribute to these differences, including scanning methods (underwater versus out-of-water, epi- or endocardial border tracing), anesthetic protocols, animal handling and levels of stress-related sympathetic activation. The lower EF in embryonic and adult zebrafish (∼35%) compared with healthy humans (≥60%) (Chahal et al., 2010) is not unexpected given that zebrafish have a relatively low-pressure circulatory system with a systolic blood pressure of ∼2 mmHg, compared with ∼120 mmHg in humans (Hu et al., 2001).

We evaluated a number of B-mode, pulsed-wave Doppler and tissue Doppler indices of systolic function and found that EF was generally associated with the largest absolute changes and smallest coefficients of variation. This is likely a result of the more reliable delineation of the ventricular chamber and volume estimations provided by speckle-tracking software in comparison with manual tracing of chamber area (for FAC) or linear dimensions (for FS). Averaging data obtained from multiple heart beats helped to account for cardiac cycle variation, and also reduced the within-animal physiological variation. Care was taken to exclude cardiac cycles affected by significant motion artefact. GLS has been shown to predict adverse cardiovascular outcome in cardiac disease (Kalam et al., 2014) and might be a more sensitive marker of early disease than EF in myocardial pathologies (Thavendiranathan et al., 2014). Hence, although we found that the coefficient of variation was often higher for GLS than EF, we suggest that GLS provides useful incremental information. In our two acquired disease models, there were parallel directions of change in all the systolic parameters measured. Thus, the combined and complementary information provided by B-mode, speckle-based deformation imaging, pulsed-wave Doppler and tissue Doppler indices might provide more robust evidence of altered contractile function, and be more useful than relying on a single parameter such as EF, particularly in borderline cases.

The high resolution permitted by the high-frequency transducer, the excellent conducting properties of water, and proximity of the zebrafish heart to the transducer allow remarkable clarity of the pulsed-wave Doppler and tissue Doppler signals, permitting an unprecedented opportunity to study diastolic ventricular function in adult zebrafish. Evaluation of diastolic properties is a key component of human cardiac function assessment, particularly in disease states in which diastolic defects precede systolic dysfunction, yet this has generally been lacking from zebrafish cardiac studies. We compared different imaging planes and found that pulsed-wave Doppler and tissue Doppler signals were best recorded in the SAX view, where the atrioventricular annulus was clearly seen and the angle between the velocity of motion and the beam of the ultrasound was minimized.

In the normal zebrafish heart, the A wave velocity was characteristically higher than the E wave, indicating that ventricular filling predominantly occurs during atrial systole. This was reflected by low E/A ratios, ranging from 0.2-0.3 in adolescent fish, down to 0.06 in adult fish, and is in marked contrast with humans, where the E/A ratio is typically ≥1.0 in healthy young adults (Dalen et al., 2010). We note that zebrafish with DTA-induced acute myocarditis were able to survive despite virtually negligible ventricular systolic function (Movie 5), suggesting that atrial contraction was the principal driver of cardiac output. Given this reliance on atrial contraction for ventricular filling, it can be predicted that atrial contractile impairment, for example, as a result of atrial dysrhythmias, would have profound hemodynamic, and potentially fatal, consequences in zebrafish.

Although high frequency echocardiography represents a major advance in assessing cardiac function, assessment of cardiac structure remains challenging. In particular, the irregular shape of the zebrafish atrium complicates evaluation of chamber size and precludes the potential application of geometric models for volume estimation. Three-dimensional imaging modalities such as micro-computed tomography and optical projection tomography might facilitate cardiac structural characterization and warrant further evaluation. As for rodent echocardiography, operator expertise is a significant variable and optimal results can only be obtained when image acquisition and data analysis are undertaken by trained experienced personnel.

In conclusion, our findings show that high-frequency echocardiography is a powerful tool for non-invasive serial assessment of cardiac function in adult zebrafish. Techniques used in human echocardiography such as pulsed-wave Doppler, tissue Doppler, speckle-based deformation imaging, and ventricular strain can now be reliably used to obtain functional data from zebrafish as young as 3 months of age. Understanding the importance of biological and technical variability, and employing techniques aimed at reducing their impact, will be crucial to future study design. This enabling technology opens up new avenues of investigation that can take advantage of zebrafish models to study cardiac regeneration as well as mechanisms, treatment and prevention of adult-onset human heart disease.

## MATERIALS AND METHODS

### Zebrafish husbandry

Zebrafish were maintained according to standard procedures (Westerfield, 2000). All experiments were performed in accordance with institutional guidelines and were approved by the Garvan Institute of Medical Research and St. Vincent's Hospital Animal Ethics Committee and the Institutional Biosafety Committee. In order to standardize fish size, zebrafish were raised at a density of 10 fish per 3 l tank containing fresh system water kept at 28°C (Hazlerigg et al., 2012). Adult wild-type zebrafish of *TE* background were evaluated (unless specified otherwise).

### Zebrafish echocardiography

#### Protocol and procedure

Fish were placed in an anesthetic chamber containing system water with low-dose tricaine mesylate at a final concentration of 0.75 mmol l^−1^ (0.02%, MS-222; Sigma-Aldrich, St. Louis, MO, USA, unless specified otherwise) to induce sedation without cessation of breathing. Anesthetized fish were transferred into a scanning chamber containing system water with the same concentration of anesthetic. The scanning chamber consisted of a glass beaker containing a weighted sponge with a groove cut into it, which served as a support for the fish while it was undergoing scanning. The fish was positioned on the sponge ventral side up, covered by ∼10 mm of water. The ultrasound transducer was then lowered gently into the water over the fish with 3 mm clearance of the body (Fig. 1). Fine adjustments of the transducer head position and movement in all three axes were achieved by mounting the transducer on to a micro-manipulator (Vevo Imaging Station, VisualSonics, Amsterdam, The Netherlands). Image acquisition was typically completed within 3 min of inducing anesthesia. After echocardiography, fish were placed in an aerated recovery chamber containing fresh system water without anesthetic and monitored. The zebrafish generally recovered within 30 s to 2 min, and there were no deaths.

#### Technical specifications and image acquisition

Echocardiography was performed using the Vevo2100^®^ Imaging System and Vevo Imaging Station (VisualSonics) equipped with a high frequency transducer (MS700D, band width 30-70 MHz, central operating frequency 40-50 MHz). Echocardiographic images were obtained in two planes: LAX (transducer positioned above the ventral surface of the fish with the transducer head in the midline, parallel to the long axis of the fish; Fig. 1A-C), and SAX view (transducer placed across the gills and heart with an orientation initially orthogonal to the LAX plane; further cranial tilting between 15-45° was required in some cases to achieve the views shown in Fig. 1D-F). Two-dimensional (B-mode) images, color Doppler, pulsed-wave Doppler and tissue Doppler signals were recorded in each view. The two-dimensional B-mode was used for measurement of heart rate, systolic function and strain analysis. B-mode specifications were: field of view 4.73×7.00 mm, high line density, medium persistence, and high sensitivity; 50 MHz transducer frame rates: 141-200 frames s^−1^, spatial resolution 75 µm (lateral)×30 µm (axial). B-mode imaging quality was further optimized by adjusting focal depth, gain, image width and depth. Color Doppler and pulsed-wave Doppler were used for the hemodynamic assessment of ventricular inflow from the atrium during diastole (Fig. 1G,J), as well as ventricular outflow to the bulbus arteriosus during ventricular systole (Fig. 1H,K). Care was taken to ensure that the direction of the ultrasound beam was parallel with the direction of flow. Maximum inflow velocity was identified as the center of the atrioventricular valve, just distal to the open valve leaflets. Similarly, for the ventricular outflow, maximal outflow velocity was obtained at the center of the bulboventricular valve just distal to the valve. Pulsed-wave Doppler specifications were: sample volume size 0.18 mm, transducer transmission frequency 40 MHz, pulse repetition frequency 25 kHz, with a 250 Hz wall filter. Tissue Doppler assessment was performed by examining the movement of the myocardium adjacent to the atrioventricular groove during the cardiac cycle, similar to the principle of myocardial tissue Doppler in humans (Fig. 1L). Specifications for tissue Doppler, used for assessment of diastolic function, were: sample volume size 0.18 mm, transducer transmission frequency 40 MHz, pulse repetition frequency 2 kHz. To account for beat-to-beat variation in ventricular measurements, at least two B-mode image sequences containing ≥5 cardiac cycles were recorded. For both pulsed-wave Doppler and tissue Doppler, ≥4 s image sequences including ≥10 cardiac cycles were recorded.

### Image analysis

Image analysis was performed using the Vevo Lab™ analysis software package v1.7.1 (VisualSonics) with the operator blinded to treatment group. Heart rate was measured from B-mode image sequences by sampling the number of heart beats in a 15 s window. For ventricular measurements, at least three representative cardiac cycles were averaged. Given that examined wild-type zebrafish showed physiological cardiac cycle variation, special care was taken to ensure that beats occurring during significant irregularity of cardiac cycle length, and also beats affected by motion artefacts, were excluded from analysis.

Ventricular chamber dimensions were obtained from B-mode images in the LAX view. EDD_BASE_ and ESD_BASE_ were obtained by measuring the distance between the inner border of the compact myocardium at the base of the ventricle at end-diastole and end-systole, respectively (Fig. 2). The corresponding ventricular EDD_LONG_ and ESD_LONG_ were then measured as the perpendicular distance from the basal diameter to the ventricular apex. VAd and VAs were defined as the area within the inner border of the compact myocardium at end-diastole and end-systole, respectively. The following estimates of ventricular systolic function were derived from diameter and area measurements: FS=(EDD_LONG_–ESD_LONG_)/EDD_LONG_; FAC=(VAd–VAs)/VAd.

Speckle-tracking analysis of ventricular wall motion was performed with the VevoStrain™ analysis software package (v1.5.0, VisualSonics) and was used to assess ventricular volumes and EF. For this analysis, the inner border of the compact myocardium was used to define the internal chamber. EF=(EDV–ESV)/EDV; cardiac output=heart rate×stroke volume (SV), with SV=EDV–ESV. The speckle-tracking software was also used to calculate ventricular wall strain (ε), defined as the change in the length (L) of a deformed object divided by the original length (L_0_)_:_ ε=L–L_0_/L_0_. Ventricular myocardial strain, displacement, strain rate, and velocity were recorded as individual values (for six separate wall segments), global values (computed average of all six segmental peak values), or peak values (highest value of any of the six segments).

To help devise a standardized ventricular measurement, we indexed EDV to body weight, body length (defined as maximal length from snout to base of tail), BMI [calculated as BMI=weight/(length)^2^] and BSA (calculated using the formula BSA=8.46×weight^0.66^, validated for fish of similar size and shape to zebrafish) (Murphy and Murphy, 1971; Niimi, 1975).

For assessment of diastolic function, we examined the following pulsed-wave Doppler signals: E wave – peak velocity of blood inflow across the atrioventricular valve during early diastole; A wave, peak velocity of blood inflow across the atrioventricular valve during atrial systole. Tissue Doppler signals obtained were: é – tissue velocity at the atrioventricular annulus during early diastole; á – tissue velocity at the atrioventricular annulus during atrial systole; ś – tissue velocity of the atrioventricular annulus during ventricular systole.

### Anesthetic comparisons

Male wild-type fish (6 months old) were anesthetized using tricaine mesylate 1.5 mmol l^−1^ (0.04%) or 0.75 mmol l^−1^ (0.02%) (Tris-buffered, Sigma-Aldrich) or 2-PE, 0.0036 mmol l^−1^ (1:2000 dilution) or 0.0024 mmol l^−1^ (1:3000 dilution, Sigma-Aldrich). Fish were immersed in anesthetic solution for 30-45 s before transfer to the scanning chamber. Heart rate and ventricular functional indices were estimated at 1, 3, 6 and 9 min after first exposure to anesthetic solution.

### Background strain comparisons

Body size, heart rate, ventricular dimensions and cardiac function were compared in male fish (*n*=10-13) from four separate wild-type strains (*TE*, *SUN*, *WIK*, *AB*), each aged 9 months. Studies were performed using the same protocol and environmental conditions.

### Diptheria toxin as a myocarditis model

The *Tg(cmlc2:CreER;bactin2:loxP-mCherry-STOP-loxP-DTA176)^pd36^* line (kindly provided by K. Kikuchi, Victor Chang Cardiac Research Institute, Sydney, NSW, Australia, and K. Poss, Duke University Medical Center, Durham, NC, USA) was used to induce acute toxic cardiomyopathy, as described (Wang et al., 2011). In this model, cardiomyocyte-specific expression of *Cre* recombinase in the presence of 4-HT (Sigma-Aldrich) induces DTA expression, resulting in marked cardiomyocyte death peaking at 7 days post-exposure. Male wild-type fish (aged 6 months) were exposed to 1.5 µM 4-HT dissolved in 100% ethanol for 12 h. Cardiac function was compared in individual fish by echocardiography before and 7 days after treatment. Following echocardiography, fish were then euthanized and their hearts explanted for morphological assessment and histology. The extent of viable heart tissue was quantified by determining the ratio of tissue expressing mCherry (viable cardiomyocytes without DTA expression) to the amount of tissue in the entire ventricle. Areas of the image that did not appear fluorescent red were considered to represent dead tissue (Wang et al., 2011). All images were analyzed using FIJI ImageJ version 1.48k (National Institutes of Health, https://imagej.nih.gov/ij/).

### Chronic anemia model

PHZ (Sigma-Aldrich) was used to induce chronic hemolytic anemia and volume overload-related cardiac remodeling in zebrafish (Sun et al., 2009). Male wild-type fish (aged 6 months), housed in individual 100 ml glass beakers, were exposed to increasing doses of PHZ every second day for an 18-day period as described (Sun et al., 2009), with the following protocol modifications implemented to improve acclimatization and prevent mortality: day 1, 1.0 µg/ml, 30 min; day 3, 1.5 µg/ml, 30 min; days 5 and 7, 1.5 µg/ml, 1 h; days 9, 11, 13 and 15, 2.0 µg/ml, 1 h. Echocardiography was performed before (day 1) and after (day 18) PHZ treatment.

### Statistics

Means±s.d. were used to report normally distributed continuous variables, whereas medians and interquartile ranges were used to report variables that were not normally distributed. Unpaired *t*-tests were used to determine differences between fish populations, and paired *t*-tests were used to compare repeated measurements in identified individual fish. Difference in means between groups at multiple time-points was calculated using repeated measures ANOVA. Intra-observer and inter-observer agreement of continuous variables between two raters was assessed (Bland and Altman, 1986). Intra-class and inter-class correlation coefficients were also calculated. Based on observations in wild-type fish, power calculations were carried out to calculate sample size for experimental models allowing the detection of up to a 20% difference in EF, with a power of 0.8 and Type I error probability of 0.05. Statistical analyses were performed using SPSS Statistics 23 (www.ibm.com/software/analytics/spss/) and GraphPad Prism 6 (www.graphpad.com/prism).
